# Ethical preparedness of data monitoring committees (DMCs) to oversee international clinical trials: a qualitative descriptive study

**DOI:** 10.1136/bmjgh-2024-015233

**Published:** 2024-08-24

**Authors:** Alex Hinga, Akram Ibrahim, Diego Vintimilla, Mickayla Jones, Lisa Eckstein, Annette Rid, Seema K Shah, Dorcas Kamuya

**Affiliations:** 1 Health Systems and Research Ethics Department, KEMRI-Wellcome Trust Research Programme, Kilifi, Kenya; 2 Mary Ann & J. Milburn Smith Child Health Outcomes, Research, and Evaluation Center, Stanley Manne Children’s Research Institute, Ann & Robert H Lurie Children's Hospital, Chicago, Illinois, USA; 3 Chicago Department of Public Health, Chicago, Illinois, USA; 4 School of Law, University of Tasmania, Hobart, Tasmania, Australia; 5 Department of Bioethics, The Clinical Center & Division of International Science Policy, Planning and Evaluation, Fogarty International Center, National Institutes of Health, Bethesda, Maryland, USA; 6 Department of Pediatrics, Northwestern University Feinberg School of Medicine, Chicago, Illinois, USA; 7 Nuffield Department of Clinical Medicine, Centre for Tropical Medicine and Global Health, University of Oxford, Oxford, UK

**Keywords:** Global Health, Qualitative study, Clinical trial, Decision Making, Descriptive study

## Abstract

**Introduction:**

A data monitoring committee (DMC) is an independent group of experts who assess the ongoing scientific and ethical integrity of a study through periodic analyses of study data. The objective of this study was to explore the extent to which the structure, membership and deliberations of DMCs enable them to address ethical issues.

**Methods:**

We conducted qualitative individual interviews (n=22) with DMC members from countries across Africa, the Americas, South Asia and the UK. We selected interview respondents through purposive sampling, managed data using NVivo (Release V.1.7) and analysed data thematically.

**Results:**

All respondents were highly experienced professionals; many (18/22) had received training in medicine and/or statistics. One respondent had academic qualifications in ethics, and four indicated that they served on DMCs as ethicists. While respondents generally felt DMCs should be required for studies that were high-risk or enrolled vulnerable populations, some were concerned about the overuse of DMCs. There were divergent views on the necessity of geographical and disciplinary representation in DMC membership, including about whether ethicists were helpful. Many respondents described a DMC member recruitment process that they felt was somewhat exclusive. While one respondent received DMC-specific training, most described learning on the job. Respondents generally agreed that study protocols and DMC charters were key guiding documents for addressing ethical issues and described DMC deliberations that often, but not always, involved consensus-building.

**Conclusion:**

This study is one of the first to consider the ethical implications of DMC structure, membership and deliberations. The potential overuse of DMCs, DMC member recruitment processes that seem somewhat insular, limited training for DMC members, and divergent approaches to deliberation may limit the capacity of DMCs for addressing ethical issues. Further research on DMC structure and processes could help enhance the ethical preparedness of DMCs.

**WHAT IS ALREADY KNOWN ON THIS TOPIC**

- A data monitoring committee (DMC) is a small expert group that is constituted to ensure participant safety and scientific integrity in ongoing clinical trials.
- DMCs are highly influential as they have access to interim and unblinded data and can recommend modification, continuation or early stopping of a trial.
- While there is extensive guidance, case study and opinion-based literature on DMC operations, relatively little is known about the ethical preparedness of DMCs.

**WHAT THIS STUDY ADDS**

- Uncertainty about when DMCs should be used, DMC recruitment strategies that appear exclusive, and limited training for DMC members could limit DMC capacity for addressing ethical issues.

**HOW THIS STUDY MIGHT AFFECT RESEARCH, PRACTICE OR POLICY**

- To enhance the capacity of DMCs for addressing ethical issues, there is a need for further analysis of which studies involve complex ethical and scientific deliberations requiring DMC input, as well as further guidance on how to ensure fair representation and inclusivity in DMC membership.

## Introduction

 Data monitoring committees (DMCs)—also referred to as data and safety monitoring boards —are small groups of experts that are independent of sponsors and investigators of clinical trials.^1^ Constituted to ensure the ethical and scientific integrity of ongoing trials,^2 3^ DMCs conduct periodic analyses of accruing interim data and make recommendations to sponsors on whether to continue, modify, pause or stop a study.^4 5^ DMCs might recommend to stop trials early in order to protect research participants from serious adverse events^6^ or accelerate the availability of treatments that have been proven safe and effective earlier than expected.^7^ When making recommendations, DMCs can consider a range of scientific, logistical and ethical issues.^8 9^ This includes weighing benefits and risks for trial participants and balancing the potentially conflicting interests of current trial participants, future patients, trial investigators and sponsors.^1 10^

Some have described DMC work as ‘motivated primarily by an ethical imperative’.^7^ DMCs are likely to encounter a wide range of ethical issues. An ethical issue can arise when an ethics principle, such as non-maleficence or scientific validity, is violated or not adequately considered (eg, participants are harmed)^11 12^ or when two or more principles are in conflict (eg, realising a public health benefit risks data confidentiality).^13^ For example, members of DMCs for neonatal intensive care trials have reported facing challenges related to balancing scientific integrity with the immediate health needs of vulnerable neonates and the expectations of their families, among other ethical issues.^14^ Additionally, DMCs have addressed whether it is ethically acceptable to continue to randomise participants to a control or placebo arm when the standard of care differs around the world and is rapidly evolving.^15 16^

DMCs and institutional review boards (IRBs) have important and overlapping roles in ethical oversight of clinical trials,^17 18^ but DMCs are generally the only oversight group with access to unblinded, emerging safety and efficacy data, giving them a unique role in ethical decision-making. Unlike IRBs and other oversight bodies that conduct ethics review, and grant ethics approvals, research licences or permits for trials to start or continue,^19 20^ DMCs are advisory bodies; trial investigators and sponsors have final responsibility for decisions and conduct of a trial.^1^ In addition, DMCs hold open session meetings with trial investigators, steering committee and sponsors to discuss administrative and wider contextual issues in a trial, such as participant recruitment rates and data quality.^8^ These ongoing discussions with others involved in a trial, coupled with DMCs independence and unique access to and review of emerging data, promote trial integrity and credibility of results.^21 22^

There are many sources of guidance for the composition and operation of DMCs. For example, DMCs are recommended for high-risk clinical trials, including randomised trials on clinical efficacy and safety of medical interventions,^5^ trials that compare rates of mortality or major morbidity,^23^ trials conducted in emergency contexts and those involving vulnerable populations.^8 24^ It is widely recommended that DMCs include members with statistical and clinical expertise while additional expertise is sometimes welcome.^8 24^ Sponsors also develop DMC charters that outline the structure, membership, deliberative processes and other issues for each DMC.^1 25^ However, DMC charters are often not publicly available.^25 26^

DMCs have a shared responsibility for ensuring the ethical conduct of ongoing trials^17 18^ and may encounter ethical issues in their work.^15^ Also, it has been suggested that a well-constituted DMC, would contribute to identification and mitigation of ethical issues in trials.^27 28^ However, the current literature on DMCs largely focuses on technical issues, such as the content of interim reports,^29^ and case studies and controversies in DMCs^30^,^32^ with limited focus on ethical issues.^15^ In addition, little empirical evidence is available regarding the structure, membership and deliberations of DMCs.^14 33^ Data from Africa are especially scant.^34^ Consequently, relatively little is known about the ethical issues faced by DMCs and the ethical preparedness of DMCs. To address this gap in the literature, we conducted a qualitative interview study with DMC members from countries across Africa, the Americas, South Asia and the UK on their ethical preparedness. Specifically, this article reports on how well the structure, membership and deliberative process of DMCs equip them to address ethical issues in international clinical trials.

## Methodology

### Research design and data tools

We used a qualitative description (QD) approach for empirical data collection and analysis^35^ to enable a rich description of events from the perspectives of individuals with directly relevant experience.^35^,^38^ The philosophical foundation for the QD approach is pragmatism with sensitivity to influences from the social, political and other contexts of research.^39 40^ Our analysis and presentation of findings involve low-inference interpretation.^36^

This study is part of a larger project on DMC ethics involving a qualitative study with DMC members, a systematic literature review and content analysis of DMC charters. The qualitative study involved interviews with DMC members to explore ethical issues faced by DMCs and ethical preparedness of DMCs. A detailed account of ethical issues faced by DMCs is reported elsewhere.^41^ This article focuses on ethical preparedness of DMCs.

For the qualitative study, we developed a semistructured interview guide in consultation with an expert advisory panel. Key domains in the interview guide included the processes and motivations for joining a DMC, views on DMC membership and deliberative processes. We also administered short written demographic questionnaires. We piloted the qualitative interview guide with other research professionals to improve clarity and reduce length. We made minor adjustments to the interview guide during data collection in line with standard qualitative methodology.^42^

### Respondent selection

As is typical for qualitative research, a purposive sampling strategy^43 44^ was used to select individuals with experience of serving in at least one DMC. We formulated the inclusion criteria and initial list of potential respondents with input from the study advisory group. The advisory group comprised two statisticians and two clinicians with extensive experience of serving on DMCs, including conducting research on DMCs and other trial oversight bodies. Based on this professional experience, the group was aware of individuals who had served on DMCs. We also selected individuals who were part of the DMC registry on the Society for Clinical Trials website. To supplement this list, we used a snowballing approach to recruit others who were recommended by participants. We confirmed eligibility when conducting interviews and did not enrol individuals who did not have experience serving on at least one DMC. Some respondents were known to members of the research team. There were no explicit refusals; however, some individuals did not respond to email requests for interviews and may be considered silent refusals.^45^ We sent an initial email request for interviews to potential participants followed by two email reminders before considering the lack of response and a refusal.

### Setting and data collection

We collected data through online (Microsoft Teams and Zoom) individual interviews for practical reasons, including COVID-19 restrictions, geographical distance and convenience for respondents. Before the interviews, an information and consent form and a semistructured interview guide (online supplemental file 1) were sent to potential respondents. The interviews were conducted by two groups of researchers within the project team; one based in the USA and the other in Kenya. Respondents gave verbal (USA led) and written consent (Kenya led). All interviews were conducted in English and audio recorded with participant consent. On average, the interviews lasted 48 min (min: 31, median: 48 and max: 72).

### Data management and analysis

Interview data were managed using NVivo (Release V.1.7) and Microsoft Excel software. Interviews were recorded in Microsoft Teams and using external encrypted digital audio recorders. Interview data and study-related documents were stored in locked cabinets and password-protected computers.

Data were analysed using the framework approach^46^ for qualitative data analysis, involving data familiarisation, coding framework development, indexing, charting, mapping and interpretation. Data familiarisation included writing a summary of the interview soon after data collection, interview transcription and data cleaning. Cleaned interview transcripts were uploaded to NVivo for coding.

An initial coding framework was developed through a highly iterative process and involved multiple coders. The process was primarily inductive, generating many codes directly from the data, but also deductive as questions from the interview topic guide were used to create additional codes. One selected interview transcript was reviewed by four members of the research team (DV, AH, DK and SKS) who each independently developed draft codebooks and then met to develop a harmonised codebook. Three research team members (DV, AH and SKS) applied the harmonised codebook to two more interview transcripts and met another time to develop the final codebook. Next, research team members (DV, MJ, AH, AI and SKS) applied the final codebook to all the interview data, which involved a primary coder and a secondary coder reading each transcript. Using NVivo and MS Excel, a framework matrix was created from the coded data, with each column representing a theme and each row representing an interviewee to allow the identification of similarities and differences in views among all study participants. Finally, our data were interpreted with reference to relevant literature. Results on the ethical issues faced by DMCs are reported elsewhere.^41^

### Patient and public involvement

Patients or the public were not involved in the design, or conduct, or reporting, or dissemination plans of our research.

## Results

We interviewed a total of 22 individuals (14M, 8F) from 9 countries with experience of serving in at least one DMC. Participants had experience as members with statistical, clinical and ethical expertise. Some had served as DMC chairs (table 1). Respondents in this study had served in DMCs overseeing a wide range of trials, most of which tested interventions against infectious diseases including malaria, tuberculosis, Ebola virus disease, HIV/AIDS, COVID-19 but also included cancer, diabetes, cardiovascular diseases and direct cash transfer interventions.

**Table 1:** Respondent demographics, background and DMC roles

Characteristic	N (%) or mean
DMC members (total N=22)	
Age, years	
40–49	7 (32)
50–59	5 (23)
60–69	4 (18)
70–79	4 (18)
80+	2 (9)
Race and ethnicity (self-identified)	
Black African	9 (41)
White non-Hispanic	11 (50)
Asian Indian	1 (5)
Mixed	1 (5)
Years of professional experience	
10–19	5 (23)
20–29	5 (23)
30–39	2 (9)
40–49	1 (5)
≥50	2 (9)
Missing	7 (32)
Gender	
Female	8 (36)
Male	14 (64)
Training (primary discipline)	
Medicine	11 (50)
Statistics and mathematics	7 (32)
Ethics	1 (5)
Biomedical science	3 (14)
Role on DMC	
Statistician	7 (32)
Clinician	8 (36)
Ethicist	4 (18)
Researcher	3 (14)
Country/region of residence	
Western Africa	3 (14)
South America	1 (5)
South Asia	1 (5)
Eastern Africa	6 (27)
UK	1 (5)
USA	9 (41)
Southern Africa	1 (5)

DMC, data monitoring committee.

We identified three major themes regarding DMC ethical preparedness: views on DMC structure, DMC membership and DMC deliberations.

### Views on DMC structure

#### Types of studies requiring DMCs

While some felt that ‘all clinical trials should have a DMC’ (R18, Clinician), most respondents felt that DMCs are important for high-risk studies, including trials with multiple sites, and studies involving vulnerable populations. They saw the value of DMCs in these types of trials to see safety signals across sites that investigators in individual sites would not be able to see well or at all. Other studies felt to require DMCs included controlled human infection (or ‘human challenge’) studies. There were also slightly different perspectives on which trial phases required DMCs. Some respondents reported serving on DMCs for phase IV trials or felt that DMCs should be used for phase I trials, but others felt that DMCs were unnecessary for early or late phase trials due to their small size or relatively low risk. As this respondent explained:

The studies [early phase trials] are so small. I think the purpose of the studies is to look at first or second use in humans [of an investigation product/intervention]… and see if you can dose escalate. The people running the trials can make such decisions. Setting up an external committee would be cumbersome and might not add much. (R3, statistician)

Additionally, respondents sometimes expressed that ethics review committees, study sponsors and regulators occasionally required DMCs beyond what was necessary. Respondents felt DMCs were established unnecessarily for studies that were minimal risk, small-scale projects or studies involving widely used products or information sharing.

This was a requirement by the sponsor, but truthfully speaking, it [the study] didn't require a DMC, but I mean they couldn't start [the study] without it… it was the ethics review committee insisting on a DMC and all [the researcher] was doing was giving information to cancer patients. (R11, ethicist)

#### DMC guidance documents

The DMC charter and the statistical analysis plan in the approved scientific protocol were described as critical documents that guide DMC operations.

I think that a good protocol, a charter outlining your obligation, stopping rules, and a data analysis plan are really critical, and if you don't have those, you're setting yourself up for trouble. (R2, clinician)

Respondents emphasised that a key component of ethical evaluation occurs prior to joining a DMC. As one respondent stated, prospective members should ‘*always review the study protocol before agreeing to serve on a DMC to make sure that [they] are comfortable monitoring [the study]*.’ (R8, statistician).

The protocol was described as useful for enabling the DMC to assess whether a study was adhering to the proposed procedures. While acknowledging the importance of a comprehensive statistical analysis plan, respondents noted that the protocol might not cover all relevant issues and that some decisions can only be made once data collection starts.

The stopping rules are perhaps not as much in detail, at least in the initial period … you're sort of looking at the protocol as it is written. But as the actual research starts, that’s when these kinds of questions come. (R14, ethicist)

The study protocol, DMC charter and other documents including consent forms and the statistical analysis plan were seen as useful to help clarify roles and resolve disputes in DMCs. However, the use of these documents varied somewhat depending on DMC members’ experience and context.

I think [disagreements] were resolved by going back to the Charter. That’s why it’s quite critical for DMCs to ensure that everything is laid out clearly in the Charter… (R18, clinician)

There haven't been that many times when I've had to go back to the Charter. There are some situations where you do need to review that, but most of the time you know the main points anyway, because they're pretty similar from trial to trial. (R10, statistician)

### Views on DMC membership

Respondents described how and why they joined DMCs and who they felt should serve in DMCs, including the ideal professional experience, geographical and disciplinary representation of DMC members. We use geographical (or local) representation to refer to the extent to which DMCs draw members from the geographical regions where a study is conducted while disciplinary representation refers to the academic training and expertise of members.

#### Processes and motivations for joining DMCs

Most respondents joined DMCs largely through recommendations and invitations from colleagues or collaborators, creating the perception of DMCs as closed clubs. In general, DMC members did not have to apply or be interviewed; accepting an invitation was often enough to become a DMC member. However, two respondents mentioned joining DMCs through a competitive process or being rejected by research regulators after nomination by study teams.

The process for joining these DMCs were generally being reached out to by study team members and expressing willingness in participating. For the smaller studies, it was basically an email invite followed by formal documentation of DMC membership. For the [Vaccine Study] I think it was a fairly more competitive process. The invitation was sent out and those interested filled forms and submitted, but there was also an extensive review of profiles of individuals to be included in the DMC. (R18, Clinician)

Motivations for joining DMCs were related to respondent’s qualifications and research interests, relationships with specific researchers and sense of responsibility to society. First, most felt they had the relevant qualifications and experiences to serve in DMCs:

I was the only pediatrician in the region for like 10 years and was also involved in some studies on Malaria and HIV/AIDS involving children. When DMCs were now needed, I think my name was floated… (R20, clinician)

Second, respondents explained that the invitations to join DMCs came from researchers or study sponsors they respected or had worked with previously. Finally, some felt that they had a duty to support, or curiosity about, high-quality research that had the potential to benefit society, as noted here:

So, what motivated me, maybe I need to be honest. One, the colleague is someone we, I totally respect. And I felt it was one of my duties…; two, due to my experience with the [vaccine], which was part of my PhD and work. Also, I was quite curious to see the end products of the research, the vaccines and how the doses would work. (R16, Biomedical Scientist)

#### Professional experience of DMC members

Most respondents felt that DMC members should be highly trained and experienced professionals because of their significant responsibilities.

But I really just want to be able to have senior members on the board who understand how complex decision making is because …. I'm turning over a lot of really critical decision making to a group of experts who I'm expecting to make decisions on my behalf… (R5, statistician)

While acknowledging the value of experience and expertise, some highlighted the need for greater efforts to recruit more members, including people with less experience, to ensure sustainability given the increasing number of studies requiring DMCs.

I think there’s need to continue to expose younger and fresh blood [to DMCs]. I think DMC is not restricted by geography, so the more people we have across the globe, the easier it will be to do trials with efficiency. We need to recast the net wide and not just look for very experienced people but look for people who have retired. Because most of the big fishes tend to be obviously incredibly busy. (R22, clinician)

#### Geographical representation

Respondents felt that local representatives are likely to have insights about the geographical and socioeconomic context of a study that would be valuable for DMC deliberations. Local representatives were described as important links between DMCs and study participants.

The local members are the eyes outside so that when things happen, they [DMC] don’t just get it through email, they hear exactly what happened. (R20, clinician)

They [country representatives] were always there, and they obviously were very important. They were integral to understanding the [local] situation… kind of a reality check. (R2, clinician)

While desirable, local representation was described as challenging to achieve, particularly for large international and multisite studies. One participant described an instance where an ethics review committee from one of the countries involved in an international study would not approve the study until a citizen of the country was included on the DMC. Respondents suggested that DMCs now generally aim to have members from the countries or regions where the studies are being conducted.

Obviously, a trial these days might have several continents and numerous countries. So, it’s not possible to have a representative from each area or each region. But I believe that there’s a conscious effort to have one or two people on the committee from outside the US and outside Western Europe. (R7, statistician)

#### Views on ethicists serving on DMCs

While respondents felt that statisticians, clinicians and clinical trial methodologists were essential for a DMC to function, they had varied and contradictory opinions on the need for ethicists on DMCs. On one end of the spectrum, some respondents felt that ethicists should not be required on DMCs. On the other end, respondents felt that all DMCs would benefit from having an ethicist on board.

First, some respondents felt ethicists should not be required on DMCs because analyses and deliberations on ethical issues were not core responsibilities of DMCs. Specifically, these respondents highlighted what they felt were DMCs’ main responsibilities and noted that ethical issues are usually addressed by ethics review committees.

I think that the purpose of the DMC is clear, it is not to make ethical decisions. It is really to make guidance on the continuity of the trial…That is not to say ethicists are not important, ethicists are critical to the conduct of research, but, you know, you can then begin to say, ‘oh what about regulatory officers, what about this?’, then you lose focus. I don’t think that you want to argue that ethicists sit on DMC. I think that there are numerous other opportunities for ethicists. (R22, clinician)

Additionally, respondents in this group felt that ethicists would have little to contribute given that most DMC discussions focus on data and clinical issues rather than ethics. They also mentioned the risk that ethics deliberations could prolong or derail DMC deliberations. Furthermore, some felt that serving in a DMC would not be the best use of skills and expertise for ethicists.

…now that will be quite a thorny issue and you know you probably want legal advice as well as ethics advice, but why do you need people with those levels of expertise to be hanging around just in case something bizarre like that happens? It’s probably not a good use of an ethicist I would think. (R15, clinician)

Lastly, respondents who felt that ethicists were not required in DMCs expressed confidence that DMCs without designated ethicists would still be able to address ethical issues, including by drawing on their ethics training and experience or consulting ethicists as needed. They mentioned that researchers often have to undergo some ethics training before being cleared to conduct research and that typically receive some form of ethics education.

Ethics is the responsibility of everybody on the committee. I teach a lecture or two on research ethics for statisticians and I know that clinicians get a good dose of ethics in clinical practice. So, all those different dimensions of ethics kind of get poured into this Data Monitoring Committee. (R7, statistician)

Second, other respondents felt that the need for ethicists and ethics expertise in DMCs was dependent on the circumstances of the study associated with a DMC. They suggested that studies involving vulnerable populations, international studies, trials involving a placebo arm, and HIV/AIDS and controlled human infection studies would particularly benefit from having a DMC member with ethics expertise. Overall, they felt that DMCs for high-risk studies needed ethicists.

Finally, some respondents felt that all DMCs should have ethicists based on their past experiences.

I think it’s extremely helpful [to have ethicists]. And it seems that the bioethicist that I have had on my committee have always been active and brought up really important questions. (R8, statistician)

One respondent described an ideal state where DMC members leveraged their diverse interests, qualifications, training and expertise to work collaboratively.

If you were to drop into a [DMC] meeting to listen, it would take you several minutes and maybe most of an hour to figure out who the ethicist was, who the clinician was and who the statistician was because we all knew enough about each other’s field to speak to the essence of the issues from that field. And that to me is perfection because now I'm not bullying my professional expertise onto the committee. (R7, statistician)

In general, those with academic training and experience in ethics endorsed the inclusion of ethicists in DMCs. Otherwise, however, there was no clear pattern in terms of support for ethicists serving on DMCs based on other respondent characteristics including gender, age, nationality or experience of working with an ethicist in a DMC. Indeed, one DMC member with extensive training and experience in ethics felt that they had been invited into DMCs because of other skills and expertise they possessed.

So, I was not getting it [DMC membership] on the basis that I had done any ethics, I was getting it because I could move things along. (R11, ethicist)

#### Training

Most respondents highlighted that they had not received formal training on how to serve in DMCs. One respondent mentioned receiving some DMC-specific training, however.

Yeah, actually, I did get specific trainings once in [City in Africa]. That was biostatistics for DMC. Ah! That was some tough maths man! (R22, clinician)

Most DMC members relied on professional experience and academic trainings to discharge their responsibilities. Most (18/22) respondents in this study had undergraduate and/or postgraduate training in medicine or statistics (table 1).

DMC members enhanced their knowledge through discussions with fellow members, reading scientific publications and attending academic talks about DMCs. Overall, training was largely described as informal, ad hoc and on-the-job; some felt that this approach to training DMC members was becoming unsustainable.

There were fewer trials at the time in the early 1970s… we had more time to think and debate amongst ourselves about the issues. We sort of taught each other what each of us knew and contributed to each other’s expertise. But now there’s such an explosion of trials and data monitoring committees, so we don't have time to learn on the job as we did in the 1970s. (R7, statistician)

### Views on DMC deliberations

With respect to the decisions facing DMCs, respondents acknowledged their important but advisory role and highlighted the responsibilities of other stakeholders, including study sponsors.

DMC is very important in determining that a study should be stopped or modified. I mean, clearly, the ethics review committee plays a role there too … if there are too many safety issues, you stop the study until you find out what’s going on. (R4, statistician)

Ultimately, everything falls on the shoulders of the investigators, the steering committee, and the sponsor, however they've shared responsibility. (R7, statistician)

Most respondents explained that DMCs generated recommendations mainly through consensus. They felt that voting in DMCs should be rare, and only when disagreements cannot be addressed through discussion.

On a DMC, you don't want to have an issue where you take a vote. But you want to continue discussing until you reach a consensus. Although sometimes it’s not possible. Sometimes there might be one or two people who don't come around. So, I think it’s important to try and reach a clear decision. (R10, statistician)

Respondents highlighted a range of ethical issues encountered by DMCs and reported how ethicists and knowledge of ethics contributed to deliberations on these issues. These included ethical issues around trial design, such as the use of placebos and social acceptability of interventions, balancing individual risks with potential public health benefits, emotional distress for DMC members and ethical issues around decisions on termination or continuation of trials.

There were a lot of ethical issues, particularly as we began to do HIV trials in developing countries where the resources were more restrained. Could you do placebo-controlled trials? Could you not? Almost always there were ethicists on those committees … and I learned a lot from them. I sort of learned some of the basic principles, and there was certainly debate and all of that, but they often would bring up important principles. (R2, clinician)

Overall, the DMC deliberative process as described by respondents typically involved data analysis and review, discussions and consensus-building. Respondents emphasised that an effective chair was crucial in guiding the DMC deliberative process.

## Discussion

We set out to explore how well the structure, membership and deliberations of DMCs support DMCs in addressing ethical issues. Our findings suggest that uncertainty about when DMCs should be used, DMC recruitment strategies and a lack of training for DMC members could limit DMC capacity for addressing ethical issues. This study adds to empirical research on DMC operations^14 33 47^ and extends the DMC ethics literature^15 17 48^ uniquely by examining the ethical implications of DMC structures, membership and deliberations. In the following, we discuss the study findings in light of existing literature and guidelines and highlight areas that require further research to enhance the ethical preparedness of DMCs.

First, the uncertainties about which studies require DMCs could lead to the overuse of DMCs and limit their capacity for addressing ethical issues. Although there are varying regulations and guidance on which trials require DMCs,^1 5 8 24^ respondents in this and other studies^33^ lacked consensus on criteria for requiring a DMC for a given study while also raising concerns about the overuse of DMCs. Given the limited resources of DMC members with expertise and training, particularly in ethics, our results suggest a need for more research on which studies should have DMCs to allocate this resource efficiently and ensure the ethical integrity of ongoing trials.

Second, perhaps related to concerns about DMC overuse, we found that there were challenges in ensuring a diverse representation of DMC members. Most guidelines welcome multidisciplinary representation in DMC membership; however, there currently is an emphasis on the selection of clinicians and statisticians^49^ over other professionals. For example, ethicists are largely recommended for DMCs overseeing clinical trials involving unusually high risks.^28^ Overall, there were varied opinions on the role of ethicists on DMCs in our study, with some respondents highlighting that the issues faced by DMCs are fairly technical and do not necessarily require ethics training. However, DMC deliberations inherently involve ethical issues, such as considering trade-offs between risks to study participants and benefits to future patients.^1 7^ Furthermore, the literature highlights a range of ethics-related issues that DMCs grapple with, including when to recommend that studies are modified or stopped, maintaining confidentiality of interim data, when new information should be shared with participants,^3 10 48 50^ the appropriate relationship between DMCs and ethics review committees^17 18^ and whether it is ethical to continue to randomise participants to placebo or control arms.^15^ Participants highlighted that an effective chair was crucial in DMC deliberations. Similarly, a review of processes in small decision-making groups suggested that DMCs chaired by experienced members with skills in discussing ethical and scientific issues would make better quality decisions.^1^

Third, the process for recruiting DMC members, as described by respondents in this study, may not be ideal for recruiting enough members for DMCs to meet increasing demand, let alone facilitate diverse representation. Individuals with limited social capital^51^ are less likely to be invited to serve in DMCs, and recruitment based on who one knows would not necessarily facilitate the inclusion of those most qualified to serve in DMCs. If those associated with a particular DMC do not know many ethicists, for instance, they may struggle to identify a qualified ethicist to serve on the DMC. A more inclusive approach to recruitment for DMCs may allow for a broader pool of individuals from different backgrounds to serve in this role and to identify and address emerging ethical issues effectively.

Fourth, DMC ethical preparedness may also be limited by a lack of training. DMC members in this study received little to no formal training on DMC work, including limited ethics training. Similarly, in a recent survey, only 8% of DMC members had received formal training, and 94% were unaware of any training programmes.^33^ However, there are a growing number of online training programmes about DMCs, and some have proposed a wide range of approaches to training DMC members, including mentorship and apprenticeships.^52^ Our study highlights the need for further analysis of what ethics training, if any, would be appropriate to enhance the capacity of DMCs to address ethical issues in ongoing studies.

Finally, respondents in this study noted that study protocols and DMC charters have a significant influence on DMC structure, membership and deliberations. These documents are used to decide whether a study is ethically acceptable enough for an individual to agree to serve on a DMC that would oversee the study. However, it has been noted that DMC charters, unlike study protocols, are not often made publicly available and accessible.^25^ Based on how critical charters were for addressing disagreements and ethical controversies, we support calls^25 26^ and ongoing efforts^53^ to make DMC charters more publicly available and accessible. This will promote transparency and sustainability of DMCs and facilitate further research on DMC preparedness for addressing ethical issues.

One limitation of this study is that we did not collect or analyse data with a focus on the type of trial or trial sponsor, which may have limited the depth of our analysis of DMC processes around specific trials and sponsors, particularly industry-sponsored trials. In addition, we relied on respondents to recall and share information on structures, membership and deliberations of the various DMCs they had served on and cannot guarantee the completeness and accuracy of all the information shared. Although we aimed to include DMC members from the Global North and South, most respondents in this study were from the USA and Africa. Future empirical ethics research on DMCs could focus on issues in specific types of trials, for example, adaptive trials and industry-sponsored trials and include DMC members from Asia and Latin America. Also, DMC experience of respondents and length of interview varied, which indicates evidence from some individuals and regions may be more prominent. However, a key strength of this study is the geographical diversity of respondents, which included members from regions that are underrepresented in the DMC literature. In addition, the research team is multidisciplinary and based in different geographical regions, with academic training and professional experience in qualitative research, global health, ethics and law. Finally, this study is one of the first to consider the ethical implications of DMC structure, membership and deliberations and demonstrates that more work in this area is needed.

## Conclusion

DMCs play important roles in ensuring the scientific and ethical integrity of research. Our findings highlight the ethical implications of DMC operations and identify areas for further research in DMC structure, membership and deliberations to enhance DMC ethical preparedness. Uncertainties about which studies require a DMC may lead to overuse or underuse of DMCs and limit the capacity of DMCs for addressing ethical issues in ongoing research. In addition, the lack of transparency about the process for recruiting DMC members raises concerns about the fair inclusion of DMC members to meet demand and to enable sound ethical deliberations. There is a need for further research to determine which studies involve complex ethical and scientific deliberations requiring DMC input. Greater attention is needed to ensure diverse representation in DMCs and enhance DMC capacity for addressing ethical issues.

## Supplementary material

10.1136/bmjgh-2024-015233 online supplemental file 1

## Data Availability

Data are available on reasonable request.
