# Supplementary material for: Ethical preparedness of data monitoring committees (DMCs) to oversee international clinical trials: a qualitative descriptive study
Source: BMJ Glob Health. 2024 Aug 24;9(8):e015233. doi: 10.1136/bmjgh-2024-015233 (PMC11404254; doi:10.1136/bmjgh-2024-015233)
Supplement: online supplemental file 1 [file bmjgh-9-8-s001.pdf]

ETHICS IN THE INTERIM: DEVELOPING NEW GUIDANCE FOR DATA AND SAFETY MONITORING OF CLINICAL TRIALS: DRAFT INTERVIEW GUIDE

Thank you for agreeing to participate in our interview of DSMB members to learn more about the ethical issues DSMBs face.

Let's begin by talking about your experience on DSMBs.

1. Please tell me a little about your current and prior experience serving on a DSMB.
    a. *Are you currently on a DSMB?*
    b. *Can you tell us how you first joined a DSMB?*
        i. *Can you tell me what interested you in serving on a DSMB?*
    c. *How many years have you served on DSMBs?*
    d. *Approximately how many DSMBs have you served on?*
    e. *What types of trials/diseases have you overseen as a DSMB member?*
        i. *Prevention vs. treatment?*
        ii. *Infectious v. chronic disease?*
        iii. *Pragmatic v. conventional?*
    f. *Have you served on DSMBs overseeing international studies?*
        i. *If the DSMBs reviewed international studies, did you feel you had the relevant expertise?*
            1. *Were there members of the DSMB from all of the countries in which it was conducted?*
    g. *What is your role (e.g., ethicist, statistician, chair, clinician, Secretary)*
    h. *Did all or most of the DSMBs you served on have a member who was an ethicist?*
2. Please tell me about your training that helps you in this role.
    a. *Have you received any training about how to serve on a DSMB? (Formal or informal)*
        i. *If yes--tell me about the training you've received.*
        ii. *If yes--when did you receive it?*
        iii. *If yes--how useful was it? What was most useful about it?*
    b. *Have you ever had any ethics training?*
        i. *If yes--tell me about the training you've received.*
        ii. *If yes--when did you receive it?*
        iii. *If yes--how useful was it? What was most useful about it?*
    c. *Have you ever had any statistics training?*
        i. *If yes--tell me about the training you've received.*
        ii. *If yes--when did you receive it?*
        iii. *If yes--how useful was it? What was most useful about it?*

3. What do you see as the most important functions of the DSMB?
    a. *Probe with the following functions if not mentioned:*
        i. *Safety monitoring?*
        ii. *Efficacy monitoring?*
        iii. *Feasibility monitoring?*
        iv. *Monitoring recruitment strategies and successes or failures?*
        v. *Ensuring trials are enrolling participants representative of the target population?*

   vi. *Overseeing integrity of the data?*
   vii. *Balancing individual v. population benefits and harms of research?*

   b. *Who should decide the DSMB's functions?*
      i. *Prompt if needed: the sponsor, the DSMB, the trial steering committee?*
   c. *Why are these functions you've identified the most important ones?*
   d. *Are there some functions DSMBs should not take on?*
      i. *If so, which ones and why?*
   e. *Have you been on trials that have monitored some, but not all, of these aspects of trials?*
      i. *Why did they do this?*
   f. *Have you ever experienced disagreement about the functions of the DSMB? Tell me more about that.*
   g. *Is there anything you've monitored on a DSMB that is not usually in the purview of a DSMB? Why was that?*

4. What kind of guidance do you think is helpful for serving on a DSMB?
   a. *Probe with the following if not mentioned:*
      i. *Stopping boundaries/rules,*
      ii. *Statistical analysis plans,*
      iii. *DSMB charter,*
      iv. *Ethical frameworks,*
      v. *Concept of equipoise*
      vi. *Ethicists/Research ethics consultation services?*
   b. *What makes this type of guidance useful or not?*
   c. *Are you aware of any ethics guidance for DSMBs—whether from an external source or specific to a trial or sponsor?*
      i. *If so, have you relied on any ethics guidance for DSMBs—whether from an external source or trial-specific?*

5. Please tell me about the biggest challenges you have faced as a DSMB member **<u>prior to</u>** the COVID-19 pandemic.
   a. *In general?*
   b. *That you would consider ethical challenges?*
      i. *Probe with:*
         1. *Making sure participants are protected,*
         2. *Balancing participant protection with population benefits,*
         3. *Considering equity in distribution of research benefits,*
         4. *Sharing or accessing confidential information,*
         5. *Relationships with other parties (e.g. sponsor, trial steering committee, DSMBs for related trials)?*
   c. *Can you tell me about some of the trials you were involved with as a DSMB member that were stopped early?*
   d. *Were there trials you were monitoring that were stopped because of the COVID-19 pandemic?*
      i. *Why were they stopped?*

        ii. *For trials that were stopped, was your DSMB consulted on how or whether to stop the trial, unblinding, or post-trial monitoring decisions?*

6. Please tell me about the biggest challenges you have faced as a DSMB member during the COVID-19 pandemic.
    a. *In general?*
    b. *That you would consider ethical challenges?*
        i. *Probe with:*
            1. *Making sure participants are protected,*
            2. *Balancing participant protection with population benefits,*
            3. *Considering equity in distribution of research benefits,*
            4. *Sharing or accessing confidential information,*
            5. *Relationships with other parties (e.g. sponsor, trial steering committee, DSMBs for related trials)?*
    c. *Have you participated in trials of interventions for COVID-19 that were stopped early?*
        i. *Can you tell me more about this experience?*

7. In dealing with these challenges, how did the DSMBs consider ethical issues?
    a. *Ad-hoc as they arose?*
    b. *With reference to pre-specified stopping boundaries/SAPs, or other guidance?*
    c. *After reviewing the ethics literature?*
    d. *After discussing with ethicists?*

8. Now that we have been in the COVID-19 pandemic for several months, what has changed (if anything) in terms of DSMB review of clinical trials?

    a. *What is working well?*
    b. *What is not working well?*
    c. *What have you learned from this experience?*

9. Based on your experience, how specific should the DSMB's statistical analysis plan be?
    a. *General statistical analysis plan or prespecified stopping boundaries?*
    b. *If the plan is at all specific, what factors should DSMBs consider to determine when not to follow it?*
        i. *Data from other trials*
        ii. *Totality of the evidence (safety and efficacy)*
        iii. *Feasibility concerns*
        iv. *Knowledge that would be useful given context in which the intervention will ultimately be given: e.g., target population, length of time intervention would be given to patients, anticipated difficulty in convincing clinicians to change behavior, anticipated future epidemiology of the disease, etc.*
    c. *If you have been on a DSMB that departed from a pre-specified plan about when to stop the trial, did you inform anyone that a boundary had been crossed? If so, who and why?*

10. What do you think about the role of an ethicist on a DSMB?
    a. *Have ethicists been included on DSMBs on which you've served?*

   b. *Should they always be included? If not, when are they most helpful?*
   c. *What have ethicists on DSMBs you've served on contributed to the team?*
   d. *Have there been downsides of including ethicists?*

11. What do you think about how IRBs/RECs, DSMBs, and regulatory agencies work together?
    a. *Have you ever served on an IRB/REC?*
    b. *Have you experienced IRBs and DSMBs working together?*
        i. *How have they worked together well?*
        ii. *Are there ways they have NOT worked together well?*
    c. *Should IRBs and DSMBs have an open line of communication, or should it all go through the sponsor?*
        i. *Why or why not?*
    d. *Should an IRB member serve on the trial's DSMB?*
        i. *Why or why not?*
    e. *Have you had interactions with regulatory agencies or regulators as a DSMB? If so, tell me more about that.*

12. If you had the ability to start from scratch and set new policies, what would you change about DSMBs to improve their ability to address ethical issues?
    a. *Should ethical issues be explicitly incorporated within statistical analysis plans and stopping boundaries?*
    b. *Should RECs/IRBs and DSMBs work more closely together? If so, how?*
    c. *Should all DSMBs be required to include ethicists?*
    d. *For international studies, should DSMBs include additional local representatives?*
    e. *Is there training that DSMB members should routinely have?*

13. Is there anything else you would like to share with us?

Thank you for taking the time to answer these questions.
